# *FHIT* loss-induced DNA damage creates optimal APOBEC substrates: Insights into APOBEC-mediated mutagenesis

**DOI:** 10.18632/oncotarget.2636

**Published:** 2014-10-31

**Authors:** Catherine E. Waters, Joshua C. Saldivar, Zaynab A. Amin, Morgan S. Schrock, Kay Huebner

**Affiliations:** ^1^ Biomedical Sciences Graduate Program, Ohio State University Wexner Medical Center, Columbus, Ohio; ^2^ Department of Molecular Virology, Immunology and Medical Genetics, Comprehensive Cancer Center, Ohio State University Wexner Medical Center, Columbus, 43210, Ohio; ^3^ Department of Chemical and Systems Biology, Stanford University School of Medicine, Standford, 94305, California

**Keywords:** FHIT, replication stress, DNA double strand breaks, APOBEC deaminase, mutations

## Abstract

APOBEC cytidine deaminase activity is a major source of hypermutation in cancer. But previous studies have shown that the TC context signature of these enzymes is not observed in sizable fractions of cancers with overexpression of APOBEC, suggesting that cooperating factors that contribute to this mutagenesis should be identified. The fragile histidine triad protein (Fhit) is a tumor suppressor and DNA caretaker that is deleted or silenced in >50% of cancers. Loss of Fhit protein activity causes replication stress through reduced Thymidine Kinase 1 expression, increased DNA breaks, and global genome instability in normal and cancer cells. Using data from The Cancer Genome Atlas (TCGA), we show that F*HIT*-low/*APOBEC3B*-high expressing lung adenocarcinomas display significantly increased numbers of APOBEC signature mutations. Tumor samples in this cohort with normal *FHIT* expression do not exhibit APOBEC hypermutation, despite having high *APOBEC3B* expression. *In vitro*, silencing Fhit expression elevates APOBEC3B-directed C > T mutations in the *TP53* gene. Furthermore, inhibition of Fhit loss-induced DNA damage *via* thymidine supplementation decreases the *TP53* mutation burden in *FHIT*-low/*APOBEC3B*-high cells. We conclude that *APOBEC3B* overexpression and Fhit-loss induced DNA damage are independent events that, when occurring together, result in a significantly increased frequency of APOBEC-induced mutations that drive cancer progression.

## INTRODUCTION

Cancer genomes are riddled with single base substitutions (SBSs), the predominant genetic alteration found in cancer cells [1–5]. Mutations in cancer genomes are not always randomly scattered, but can occur in discreet patterns. Large-scale sequencing efforts have uncovered patterns of spatially close and strand-coordinated mutations that likely occur at the same time [6–8]. Discovery of the molecular mechanisms facilitating accumulation of these SBSs, and selective expansion of cells carrying them, is clarifying the processes of accumulation of mutations that favor cell survival, proliferation, and invasiveness.

Knowledge of endogenous enzymatic sources of mutations has been elusive, but cancer genome and exome sequencing endeavors have exposed striking cancer-specific mutation signatures that suggested previously unconsidered candidates. The most pervasive of these signatures, observed in at least 16 of the most common forms of cancer [6], is defined as an abundance of C > T and/or C > G mutations at TC dinucleotides (mutated base underlined) [6–10]. Because of the specificity for TC motifs, this signature has been attributed to the APOBEC family of cytidine deaminases, enzymes that are unable to edit double-stranded DNA (dsDNA) and exhibit exquisite specificity for single-stranded DNA (ssDNA) substrates [11]. The frequency of SBSs reportedly due to APOBEC enzymes is positively correlated with expression level of APOBEC3A (A3A) and/or APOBEC3B (A3B) mRNA in cancerous cells and tissues [13–18]. Thus, A3A and A3B are the likely family members responsible for these signature mutations. Induction of dsDNA breaks has been shown to stimulate the production of APOBEC-mediated mutations [7, 12], and APOBEC TC context mutations often colocalize with rearrangement breakpoints in cancer cells. These mutations are also frequently found within known cancer driver genes [4, 10, 13, 14].

In spite of the evidence for APOBEC enzymatic activity in generating the TC motif hypermutation seen in cancer, previous analyses reveal that numerous tumors with high A3B mRNA expression exhibit very few mutations [13, 14], suggesting that APOBEC activation alone is not sufficient cause for the hypermutation profile. Another requirement is availability of APOBEC target ssDNA. ssDNA increases in cells as a result of transcription, replication, and recombination. When these processes encounter stress, abnormally long ssDNA regions accumulate and dsDNA breaks form, leading to production of ssDNAs during repair [19, 20]. For example, replication stress can cause uncoupling of polymerase and helicase activities due to an obstruction in the replicating DNA, which can lead to abnormally long stretches of ssDNA. This ssDNA accumulation recruits repair proteins to remove the obstruction. During this process the stalled forks are cleaved by endonucleases to produce dsDNA breaks and repaired by homologous recombination, which involves resection of dsDNA break ends by exonucleases, a process that creates ssDNA [19, 20]. Frequently, this type of damage activates one of the cell cycle checkpoints and arrests the cell cycle, providing time for faithful DNA repair or induction of cellular senescence [21]. If the damage is too extensive, cells instead undergo apoptosis [22].

We have recently described a source of endogenous DNA replication stress and dsDNA breaks that neither activates cell cycle checkpoints nor induces apoptosis; in other words, this damage is checkpoint “blind.” Through ongoing research on functions of common chromosome fragile site (CFS)-associated genes, we found that loss of Fhit protein expression causes a nucleotide imbalance, specifically a decrease in dTTP pools due to lack of sufficient thymidine kinase 1 (TK1) protein activity. The nucleotide imbalance leads to replication fork stalling, collapse, and dsDNA breaks [23, 24]. This checkpoint-blind replication stress leads to downstream global chromosomal instability [25]. Among the various types of genome instability observed in *Fhit^−/−^* mouse tissues and derived cell lines, we observed large increases in SBSs, including robust increases in C > T mutations (Paisie et al., in review). Alterations at the FRA3B CFS, centered within the *FHIT* gene locus, leads to partial or full loss of *FHIT* gene and protein expression in >50% of sporadic cancers [26–29], many of which exhibit APOBEC signature mutations [6, 14].

Because of the dependence of APOBEC enzymes for ssDNA substrates and the observation that many cancers with the APOBEC signature are Fhit negative, we hypothesized that Fhit protein loss creates an environment of ongoing DNA damage ripe for APOBEC hypermutation. To test this hypothesis we analyzed The Cancer Genome Atlas (TCGA) data for lung adenocarcinoma, tumors which exhibit frequent loss of Fhit expression and display the APOBEC signature, and determined that *FHIT*-low/*APOBEC*-high lung cancers exhibit the highest numbers of C > T and C > G mutations with the TC dinucleotide signature. *In vitro* Fhit-deficient/A3B-high cells displayed an increased frequency of C > T mutations in an amplified *TP53* DNA fragment; inhibition of Fhit loss-induced replication stress and dsDNA breaks *via* thymidine supplementation decreased the mutation frequency below that of Fhit-normal/A3B-high cells. Fhit protein loss occurs in the earliest prenoplastic lesions of multiple types of cancer [26–29]. Thus, we have proposed that Fhit loss-induced replication stress, and resulting dsDNA break accumulation in preneoplasias, initiates the genome instability that can lead, through progressive waves of APOBEC-mediated mutation and clonal expansion, to sporadic cancers.

## RESULTS

### APOBEC mRNA overexpression shows correlation with increased mutations

**Figure 1:**
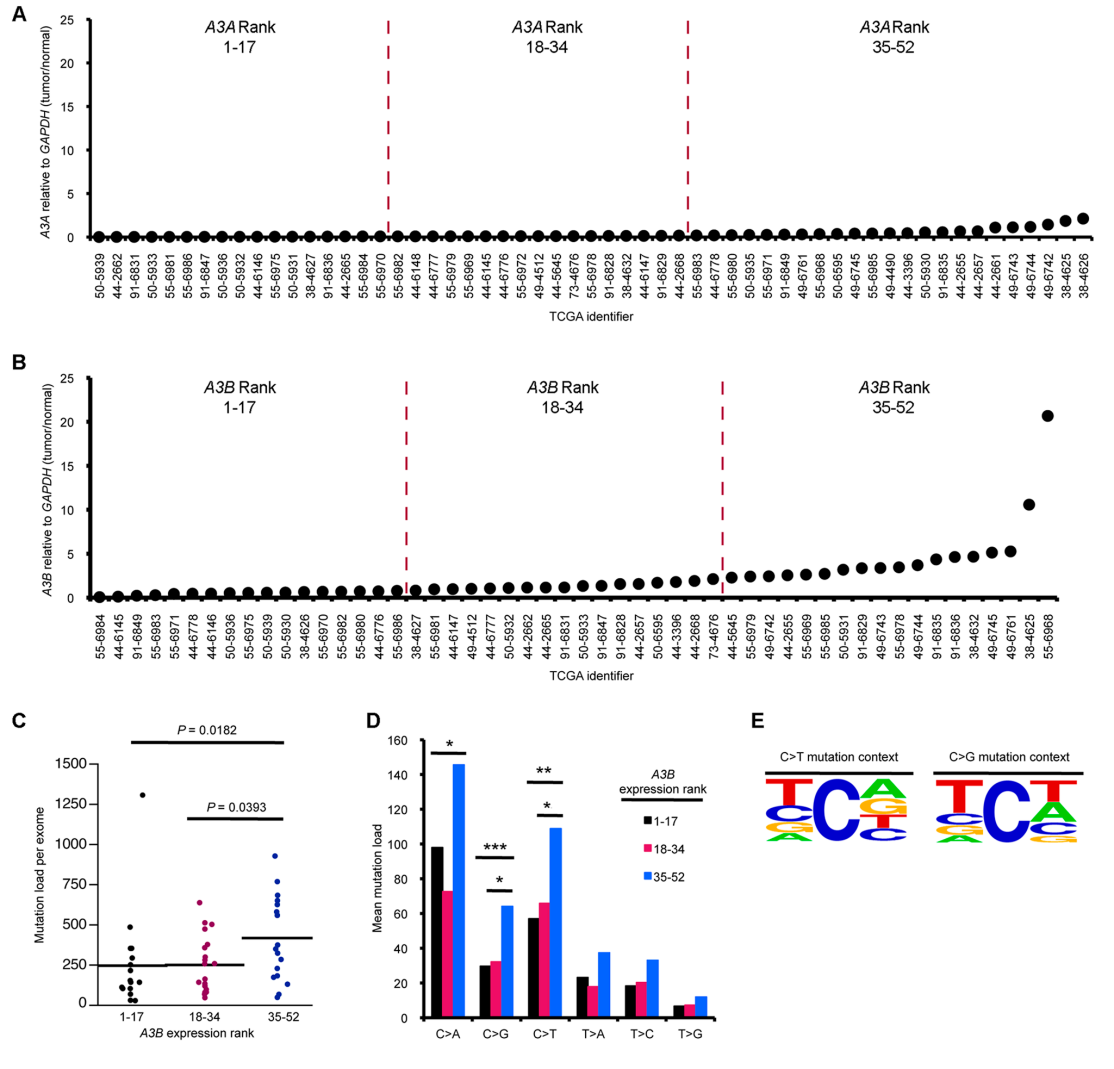
APOBEC overexpression correlates with hypermutation in lung adenocarcinoma RNA-seq and DNA-seq mutation data sets for lung cancers were downloaded from TCGA. *A3A* **(A)** *and A3B* **(B)** mRNA levels (tumor/normal) in the indicated TCGA lung adenocarcinoma samples. Tumor DNAs were grouped into low, middle and upper thirds based on *A3B* mRNA expression, such that tumors 1–17 express the lowest levels of A3B mRNA while samples 35–52 express the highest levels. **(C)** Mutation load per exome in tumor DNAs with different *A3B* mRNA expression levels. **(D)** Mean mutation load for specific transition and transversion mutations in *A3B* groups. **(E)** 5′ & 3′-flanking nucleotides of C > T transitions and C > G transversions in lung adenocarcinoma DNAs. Frequency plots were generated using www.weblogo.berkeley.edu [35]. Font size is proportional to nucleotide frequency and nucleotides at the top of the column occur most frequently. P values in (C) and (D) are from Mann-Whitney U test. (*P < 0.05, **P < 0.02, ***P < 0.009).

Using publically available TCGA RNA expression and exome sequencing datasets, we first determined *A3A* and *A3B* mRNA expression levels in each tumor sample in order to then examine the correlation of *A3B* and *A3A* mRNA overexpression (*A3B-, A3A*-high) with SBS mutations in lung adenocarcinomas. Only *A3B* was overexpressed in this patient cohort and thus we continued our analysis with A3B only (Figure 1A, B). With the expression datasets, we stratified the tumor cohort, by mRNA expression levels of *A3B*, into three roughly equal groups and compared mutation loads among tumors with low, intermediate, and high *APOBEC* expression. Increasing *A3B* mRNA levels correlated with increased total mutation load per exome and an increase in C > A, C > G, and C > T mutations (Figure 1C, D). C > T mutations were the dominant SBS found in this tumor cohort (Figure 1D). C > A mutations, a signature of environmental exposure to tobacco carcinogens, are common in lung cancer DNAs [6]. Examination of the local sequence context for C > T transitions and C > G transversions in the samples showed that these SBSs are largely focused at nucleotides that match the preferred APOBEC target TC dinucleotide context (Figure 1E), suggesting that A3B is a major enzymatic source of mutation in this tumor cohort. However, a fraction of the lung cancers with high *A3B* mRNA had very few mutations per exome; 33% of A3B-high tumors exhibited fewer than 242 mutations per exome, the average mutation load per exome for A3B-low tumors. This suggests that APOBEC overexpression is not sufficient to induce hypermutation.

### *FHIT*-low/*APOBEC*-high tumors display the highest frequency of SBSs

**Figure 2:**
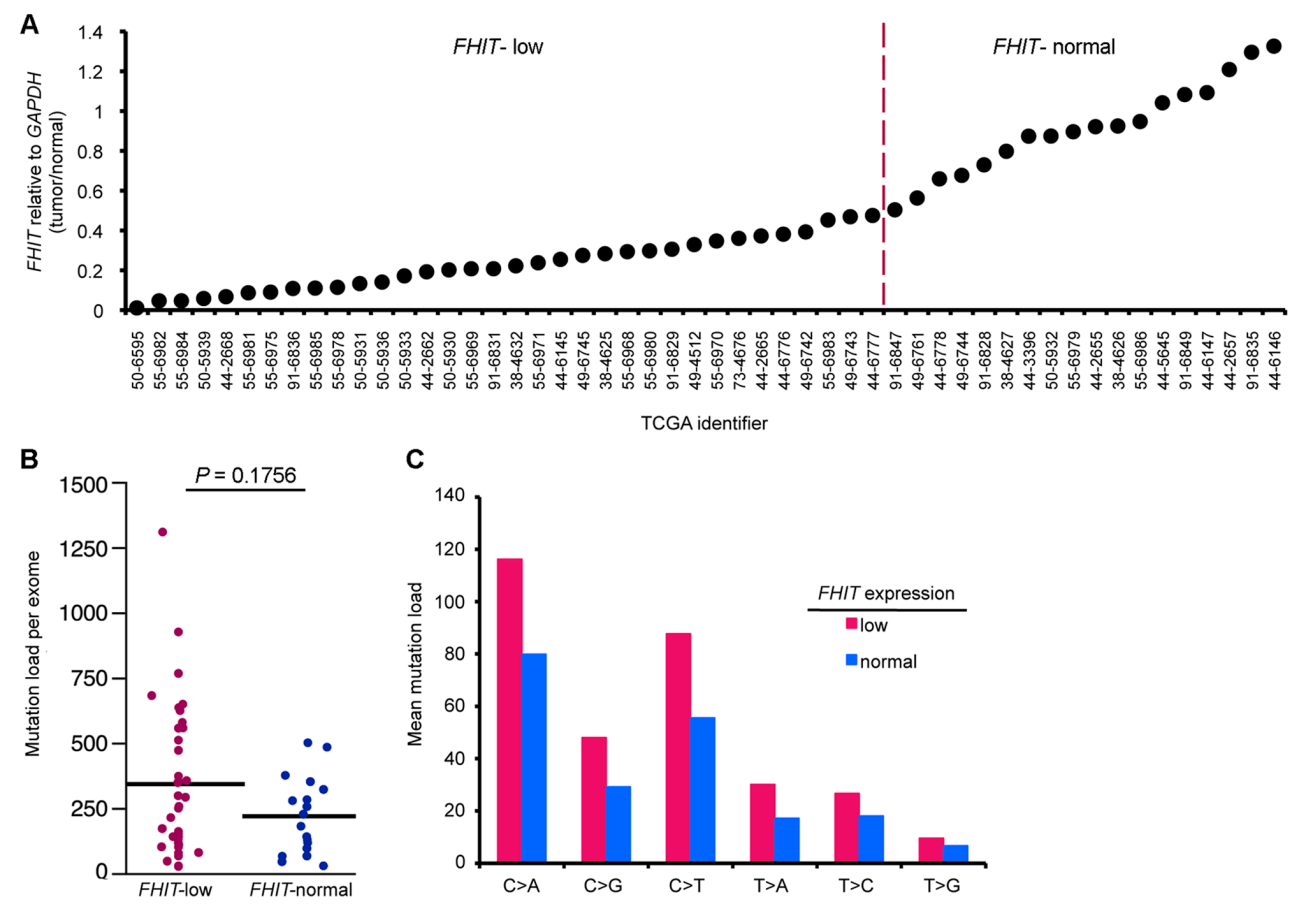
Low FHIT expression alone correlates with modest hypermutation in lung adenocarcinoma DNAs **(A)** *FHIT* mRNA levels (tumor/normal) in the indicated TCGA lung adenocarcinoma samples. Tumor DNAs were grouped into *FHIT-*normal and *FHIT*-low lung tumor DNAs (>0.5 T/N-normal, <0.5 T/N-low). **(B)** Mutation load per exome in *FHIT-*normal and *FHIT-*low tumor DNAs. **(C)** Mean mutation load for specific point mutations in *FHIT-*normal and *FHIT-*low tumor DNAs. P values in (B) are from Mann-Whitney U test. (*P < 0.05, **P < 0.02, ***P < 0.009).

**Figure 3:**
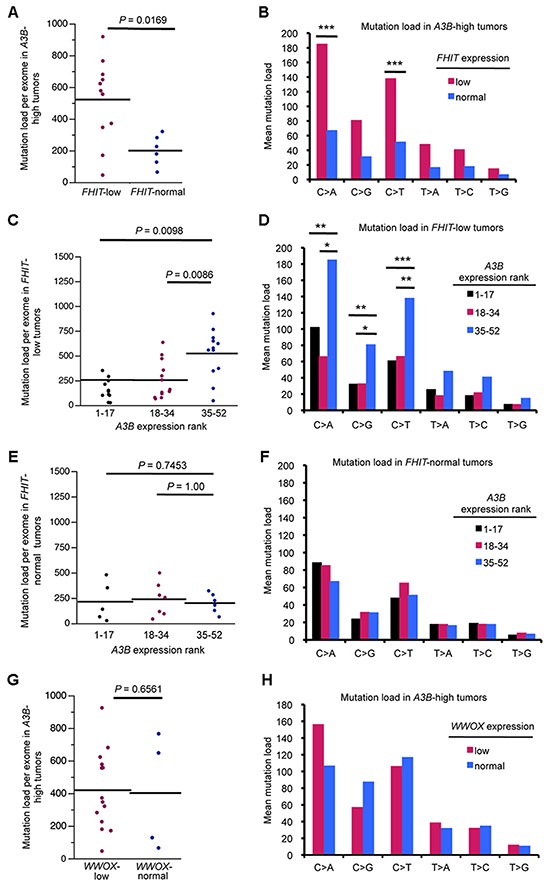
*A3B-high/FHIT*-low lung adenocarcinoma DNAs exhibit pronounced hypermutation **(A)** Total mutation load per exome in *FHIT-*normal and *FHIT*-low lung tumor DNAs (>0.5 T/N-normal, <0.5 T/N-low) in *A3B*-high tumors. **(B)** Mean mutation load for specific point mutations in *A3B*-high lung tumor DNAs with normal and low *FHIT* mRNA expression. **(C)** Mutation load per exome in *FHIT-*low tumor DNAs stratified by *A3B* mRNA, grouped into lower, middle and upper thirds. **(D)** Mean mutation load for specific point mutations in *FHIT-*low tumor DNAs stratified by *A3B* mRNA. Mutation load per exome **(E)** and mean mutation load for specific point mutations **(F)** in *FHIT-*normal tumor DNAs stratified by *A3B* mRNA. **(G)** Total mutation load per exome in *WWOX-*normal and *WWOX*-low lung tumor DNAs (>0.5 T/N-normal, <0.5 T/N-low) in *A3B*-high tumors. **(H)** Mean mutation load for specific point mutations in *A3B*-high lung tumor DNAs with normal and low *WWOX* mRNA expression. *P* values in all panels are from Mann-Whitney U test. (*P < 0.05, **P < 0.02, ***P < 0.009).

Because a subset of *APOBEC*-high tumors was resistant to APOBEC-induced mutations, we hypothesized that these tumors may produce a low level of dsDNA breaks and ssDNA *vs* those enriched with mutations. Fhit loss has been shown to increase replication stress and DNA breaks in the types of cancers displaying APOBEC signature mutations, so we next determined if stratifying the same lung cancers by *FHIT* mRNA levels, in addition to *APOBEC* levels, might suggest an explanation for the lack of mutations in some *APOBEC* high tumors and enrichment of mutations in others. Tumors with *FHIT* expression level <0.5 (tumor/normal) were considered *FHIT*-low (Figure 2A), as it is known that loss of one *FHIT* allele causes haploinsufficiency for previously studied Fhit functions [30]. *FHIT*-low lung adenocarcinoma DNAs displayed a moderate, but not significant, increase in total mutation load per exome and mutations at individual nucleotides (Figure 2B, C). The individual nucleotide mutation pattern in *FHIT*-low tumors mimicked the increase in C > A, C > G, and C > T seen with *A3B* overexpression but to a more modest level (Figure 2C). We then divided the *A3B*-high tumors into two groups: *A3B*-high/*FHIT-low* and *A3B*-high/*FHIT*-normal. *A3B*-high tumors with low *FHIT* showed significant increases in total mutations per exome and in C > A and C > T mutations, as well as increased C > G mutations (Figure 3A, B). This suggests cooperation of low *FHIT* expression with *A3B* overexpression to allow pronounced hypermutation. Alone, both *A3B* overexpression and *FHIT* loss lead to increased mutation frequency. However, hypermutation by *A3B* is more significant in *A3B*-high/*FHIT*-low tumors than in tumors with *A3B* overexpression alone. When examining total mutation load per exome in *FHIT-*low *cancers*, increased *A3B* expression was associated with a significant increase in both total mutation load and C > A, C > G, and C > T mutations (Figure 3C, D). However, when examining lung cancers with normal *FHIT* expression level (*FHIT*-normal), this association was lost (Figure 3E, F); ie, *A3B* overepxression had no effect on total mutation load or individual point mutations in tumors with normal *FHIT* expression levels. This suggests that DNA in cells that have lost Fhit caretaker function is more sensitive to APOBEC-induced mutagenesis, while genomic DNA from *FHIT* positive cells are protected from APOBEC activity. Because a positive association between *A3B*-high/*FHIT*-low tumor status and mutation load was seen in the lung adenocarcinoma data (R = 0.5076, P = 0.00218), we also analyzed tumor DNAs from a heterogenous breast cancer TCGA cohort to determine the mutation load in *A3B*-high/*FHIT*-low tumor DNAs (not shown). Due to the mixed subtype nature of the breast tumor cohort, the clear trend toward increases in mutations due to the *A3B*-high/*FHIT*-low status did not reach significance. We believe that a study including more breast cancers stratified by specific tumor subtype will provide important information about association of *A3B* hypermutation not only with Fhit loss but also with specific clinical features. For example, HER2-overexpressing breast cancers have demonstrated enrichment for APOBEC mutagenesis [14] and both HER2 positive and Triple Negative breast cancer subtypes exhibit reduced Fhit protein expression in most tumors [31].

### Expression level of a second fragile gene, *WWOX*, does not correlate with APOBEC hypermutation in lung tumors

To differentiate the consequences of reduced gene expression of specific CFS-encoded genes, we repeated our lung cancer analysis, focusing on another tumor suppressor gene product, *WWOX*, expression of which is highly reduced in a large fraction of lung cancers. Although Wwox protein is a tumor suppressor, it has not been shown to have genome caretaker function, as is the case for Fhit. In the TCGA lung tumor cohort, tumors with low *WWOX* (<0.5 tumor/normal) expression displayed no increase in total mutations per exome or increased C > A, C > G, or C > T mutations indicative of APOBEC hypermutation, neither by stratification by *WWOX* alone (Supplementary Figure 1), nor by stratification by *WWOX* level in *A3B*-high tumors (Figure 3G,H). This indicated that *WWOX* expression level does not affect the ability of A3B to mutate genomic DNA. This suggests that loss of CFS gene products in general does not affect APOBEC activity but rather that loss of Fhit caretaker function is key to enhancing APOBEC mutagenesis.

### Fhit loss-induced DNA damage and A3B overexpression cooperate to cause mutations *in vitro*

**Figure 4:**
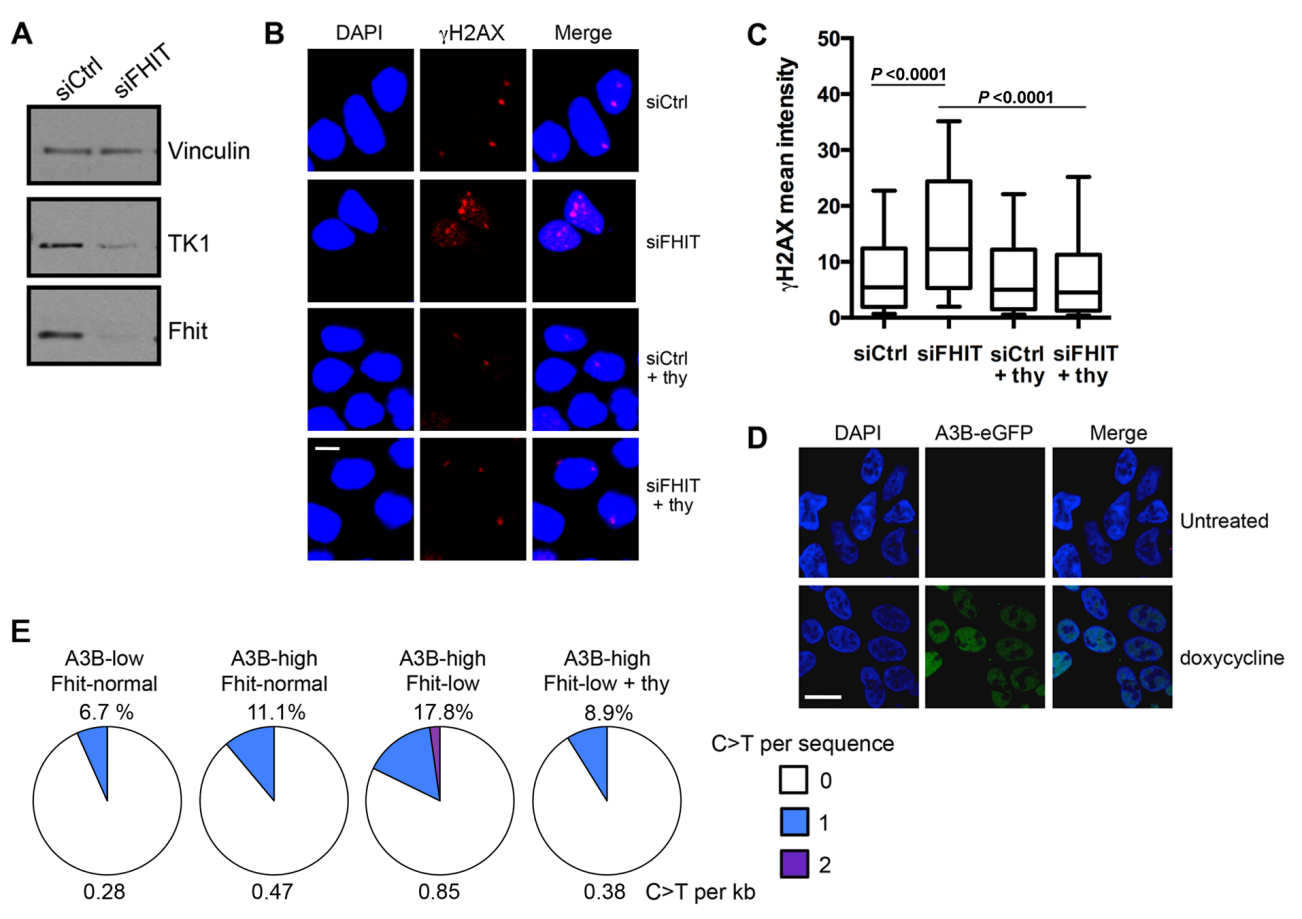
TP53 DNA amplified from A3B-high/Fhit-low TREx 293 cells exhibit an increased frequency of C > T mutations **(A)** Western blot analysis of TK1 expression in si*FHIT* transfected TREx-293 cells. Representative blot of >3 independent experiments is shown. **(B)** Representative fields of TREx-293 cells imaged for γH2AX 2 days after si*FHIT* siRNA transfection (40x), bar 5 μm. **(C)** Box plots for mean fluorescence intensities of γH2AX for data pooled from three independent experiments. **(D)** Representative fields of cells imaged for A3B–eGFP, 3 days after induction with 1 μg/ml doxycycline (100x), bar 10μm. **(E)** Pie charts illustrating the frequency of C > T mutations per clone in designated conditions of expression. C > T mutations in *TP53* detected by sequencing cloned, nested PCR products, 3 days after A3B induction and *FHIT* knock-down (*n* = 45 sequences per condition). C > T mutations occurring at GpC dinucleotides were excluded to avoid inclusion of mutations due to PCR error [37].

To validate the *in silico* correlations found in our TCGA analysis, we sought to determine if Fhit-low/A3B-high protein expressing cells accumulate C > T mutations in *TP53* DNA more frequently than Fhit-expressing cells. Both Fhit loss and A3B overexpression have previously been associated with mutated p53 [13, 25]. For our *in vitro* model system we used a TREx-293 clone [13] that stably expresses a doxycycline-inducible A3B-GFP gene (Figure 4D), graciously given to us by the Reuben Harris lab. 293 cells normally express moderate levels of Fhit, so we silenced Fhit with siRNA to produce A3B-high/Fhit-normal cells and A3B-high/Fhit-low cells (Figure 4A, D). In this model, *FHIT* silencing led to a decrease in TK1 protein expression (Figure 4A); Fhit loss-induced down-modulation of TK1 expression causes nucleotide imbalance and subsequent replication stress [23]. *FHIT* silencing also led to an increase in γH2AX foci (Figure 4B, C), indicating increased dsDNA breaks in this model due to Fhit loss and subsequent TK1 reduction. Supplementation of Fhit-silenced cells with 10 μM thymidine, the substrate for TK1, which has previously been shown to rescue cells from Fhit loss-induced replication stress [23], decreased the mean fluorescence intensity of γH2AX (Figure 4B, C), indicating rescue from Fhit loss-induced DNA damage. We then performed nested PCR amplification for a 235 bp*TP53* DNA fragment using DNA from cells with the following conditions of protein expression: A3B-low (uninduced)/Fhit-normal, A3B-high (induced)/Fhit-normal, and A3B-high (induced)/Fhit-low (silenced). The PCR products were then cloned into pcDNA3 plasmid and DNAs of individual clones were sequenced. Induction of A3B alone (A3B-high/Fhit-normal cells), caused an increase in the percent of clones displaying C > T mutations from 6.7% to 11.1% over A3B-low/Fhit-normal cells, with an average of 0.47 C > T mutations per kb (Figure 4E). A3B-high/Fhit-low clones showed the highest frequency of C > T mutations, with 17.8% of clones exhibiting these mutations (Figure 4D). This corresponded to an increase to 0.85 mutations per kb (Figure 4E). In order to confirm that, mechanistically, Fhit loss-induced dsDNA breaks were responsible for the increased incidence of C > T mutations, we supplemented A3B-high/Fhit-low cells with 10 μM thymidine in order to rescue the cells from Fhit loss-induced replication stress and DNA damage. Following thymidine supplementation, the percentage of A3B-high/Fhit-low clones with C > T mutations decreased to 8.9% (0.38 mutations per kb), less than the percentage for A3B-high/Fhit-normal clones (Figure 4E). These *in vitro* experiments confirm the correlations in the TCGA data and support the hypothesis that Fhit loss and A3B overexpression cooperate to cause hypermutation that could facilitate cancer initiation and/or progression.

## DISCUSSION

Most cancers, whether familial or sporadic, exhibit genome instability, which underlies development of suppressor and oncogene mutations that drive progression to lethal cancer stages [1, 2]. The results of this study suggest a logical pathway to the nucleotide context-specific hypermutation genotype observed in many cancers. The hypothesis stems from several parallel findings: **1)** that loss of the fragile *FHIT* gene product, Fhit, which occurs early in development of most human cancers, results in replication fork stress due to dTTP imbalance, production of dsDNA breaks and thus ssDNA during DNA repair, and extensive genome instability in all *in vitro* and *in vivo* models tested [23–25]; **2)** that the activation/overexpression in cancers of A3B and/or A3A cytidine deaminase enzymes, known previously to be involved in C > T mutations in ssDNA substrates of viral genomes, could be the cause of the hypermutation observed in many of the most common cancers [6, 14]. Thus, we sought to demonstrate Fhit loss-induced DNA damage as a cooperating factor necessary to enhance A3A/B-induced hypermutation.

Analysis of TCGA datasets for lung adenocarcinoma revealed that the APOBEC signature was prevalent in this type of cancer, for both C > T and C > G mutations. *A3B*, but not *A3A*, was overexpressed in this cohort. Tumors with both low *FHIT* expression and *A3B* overexpression showed a significantly higher number of C > T and C > G mutations than those with *A3B* overexpression alone. Furthermore, in *FHIT*-normal tumor DNAs, *A3B* overexpression had no effect on the frequency of total mutations or C > T/C > G mutations, indicating that functional Fhit protein impedes APOBEC-induced hypermutation. We further showed *in vitro*, that silencing Fhit expression in A3B-overexpressing cells increased the occurrence of C > T mutations in the *TP53* gene, validating the association between Fhit loss and A3B mutations in the TCGA datasets. The increase from 0.47 mutations/kb in A3B-high/Fhit-normal clones to 0.85/kb in A3B-high/Fhit-low clones equals 378 additional A3B-mediated mutations/Mb. This mutation frequency was decreased following thymidine supplementation, supporting the claim that it is Fhit loss-induced replication stress and subsequent dsDNA breaks that increase A3B-mediated mutagenesis. Interestingly, C > A mutations were also enriched in *A3B*-high/*FHIT*-low lung adenocarcinomas. *FHIT* gene deletions occur very frequently in response to tobacco carcinogens; thus, this mutation signature is likely a result of carcinogen exposure due to smoking.

**Figure 5:**
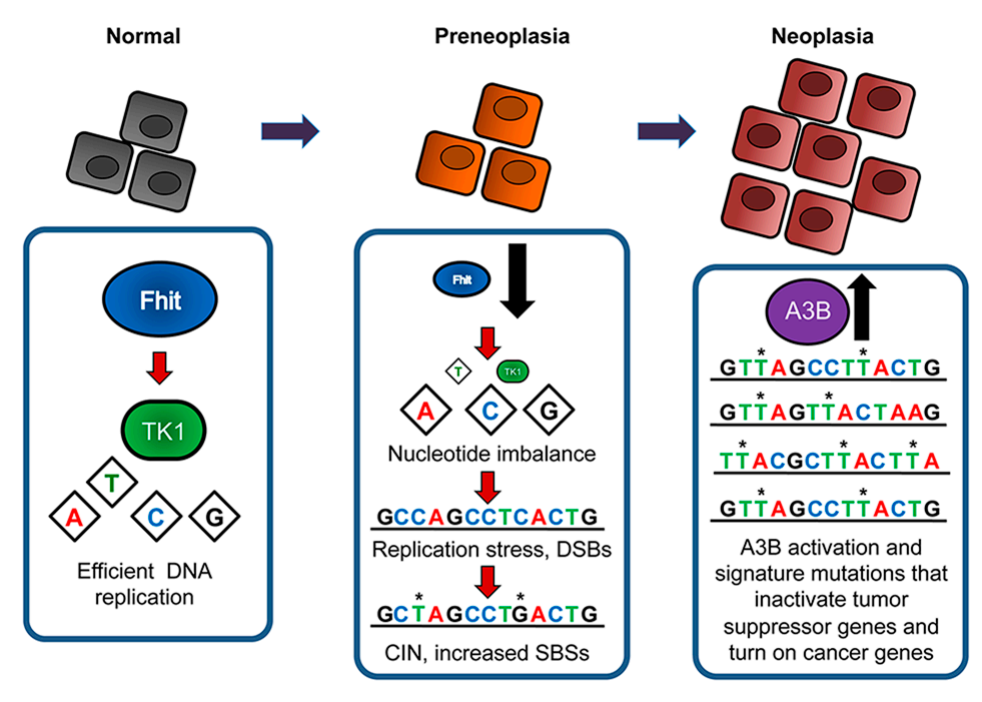
Fhit loss-induced DNA damage model for APOBEC-mediated hypermutation In normal cells, Fhit protein expression positively regulates thymidine kinase 1 (TK1) expression, thus ensuring sufficient intracellular dTTP pool levels for efficient DNA replication. Deletions or other alterations in *FHIT* alleles occur due to FRA3B fragility causing loss of Fhit protein expression in early preneoplastic lesions. Subsequent decreases in TK1 and dTTP pools causes checkpoint-blind replication stress, resulting in DSBs. DSBs promote the acquisition of chromosomal instability (CIN), including single base substitutions (SBSs). When A3B becomes activated, the moderate level of SBS mutations in Fhit negative cells becomes exacerbated due to mutagenic effects of A3B on damaged DNA substrates, thus increasing the likelihood of mutations in tumor suppressor genes and/or oncogenes that are observed in neoplastic cells.

These findings imply that loss of Fhit function is a limiting step for the formation of the APOBEC-mutational signature. Loss of Fhit caretaker function causes spontaneous replication stress [23–25], generating transient dsDNA breaks with each round of DNA replication and increased ssDNA levels during DNA repair of these breaks. Upon A3B overexpression, cytidine nucleotides within these areas of ongoing dsDNA repair would be deaminated by A3B, leading to increased genomic uracil. Removal of genomic uracil has been shown to produce clusters of C > T and/or C > G mutations after subsequent rounds of DNA replication and/or aberrant genomic uracil excision repair activities [32, 33]. Aberrant uracil excision repair occurs when base excision repair activities are ‘hijacked’ by the mismatch repair pathway [34], which requires more DNA resection than BER and consequently more exposed ssDNA to act as a template for A3B. This model for hypermutation due to synergism of Fhit loss and A3B overexpression is outlined in Figure 5, and is likely to apply to multiple types of cancers.

The discovery of cytidine deaminase enzymes such as A3A and/or A3B as a major source of mutation is ground-breaking not only in revealing a mechanism of somatic hypermutation in cancer genomes, but in revealing possible therapeutic targets for common forms of cancer, targets that spare normal cells because: 1) APOBEC enzymes are oftentimes over-expressed in cancer cells and not normal cells [13] and 2) they are non essential enzymes, so transitory or temporary inhibition of their activity may have little effect on normal cells [9]. In light of our findings, it will be useful to stratify cancer cohorts by both *FHIT* and *A3B* expression to more accurately predict which cancers are most susceptible to APOBEC-induced hypermutation. Further analysis of larger patient cohorts and more types of cancers, especially those with associated clinical features available for correlative studies, could tell us if APOBEC activity is associated with specific clinical features.

The current study shows that DNA damage/repair-generated APOBEC substrates are a prominent feature of Fhit loss and are major facilitators of A3B activity. Might there be other sources of damage-induced substrate DNAs during cancer development, such as mutated familial caretaker genes, oncogene mutation and possibly even cancer therapeutics? In response to cytotoxic and radiation treatment many cancer cells are eliminated, but some cells, including cancer stem cells, escape DNA damage-induced death and become resistant to further treatment. These resistant cells likely produce enhanced ssDNA levels due to DNA damage caused by the therapy. If this therapy-induced abundance of ssDNA occurs in cancers with activated A3B, A3B hypermutation may contribute to development of resistance to cancer therapy, a major hurdle to conquering this disease, highlighting the importance of clues to DNA mutation mechanisms and underscoring the importance of correlating APOBEC activity with synergizing factors and clinical features in order to better approach cancer prevention and treatment strategies.

## MATERIALS AND METHODS

### Cell lines, mouse tissues and reagents

T-REx-293 clones expressing doxycycline-inducible A3B-eGFP were obtained from the Harris lab [13] and were cultured in DMEM with 10% FBS, 1% pen/strep, 50 μg/ml hygromycin, and 20 μg/ml blasticidin. A3B-eGFP was induced with 1 μg/ml doxycycline for 3 days.

### Analysis of TCGA sequence data

Somatic mutations and RNA-seq expression data for lung and breast tumor and corresponding normal samples were downloaded from The Cancer Genome Atlas (TCGA) Data Matrix. Gene expression values were mined from RNA-seq data sets. *APOBEC3A (A3A), APOBEC3B (A3B), FHIT,* and *WWOX* expression values were normalized to the expression of *GAPDH* for each tumor and normal sample. Relative *A3A, A3B, FHIT* and *WWOX* expression levels were determined *via* comparison of tumor samples to corresponding normal samples (tumor/normal). Samples were divided into 2 groups for *FHIT* expression (and *WWOX*): low (<0.5) and normal (>0.5). Samples were also stratified based on *A3B* expression levels and divided into three roughly equal groups so that the group with the lowest assigned numbers express the lowest level of gene expression. Duplicate mutations were removed and the total number of mutations per exome was determined as well as the number of mutations at individual nucleotides. Mutation local trinucleotide context sequences for C > T transitions and C > G transversions were used to determine mutation context *via* www.weblogo.berkeley.edu and displayed as weblogo motifs [35].

### PCR and clone sequencing

DNA was collected from T-REx-293 clones [13] expressing doxycycline-inducible A3B-eGFP, with and without *FHIT* silencing *via* siRNAs (ON-TARGET plus siRNAs, Thermo). Outer amplification for nested PCR for a 235 bp portion of *TP53* was performed as follows: 95°C for 5 min followed by 30 cycles (95°C for 1 min, 60°C for 30 sec, 68°C for 30 sec). Inner PCR, with 1/25 of the previous reaction as DNA input, was performed using the following program: 87°C for 5 min followed by 30 cycles (87°C for 1 min, 60°C for 30 sec, 68°C for 30 sec). The PCR products were analyzed by gel electrophoresis with ethidium bromide, purified (Machery-Nagel), sticky-end cloned into pcDNA3 vector, sequenced with T7 primer (IDT), and aligned and analyzed with Finch TV software (Geospiza Inc.).

### Western blot analysis

Cells were lysed with RIPA buffer (Thermo Scientific) supplemented with Halt Protease Cocktail Inhibitors (Thermo Scientific). Proteins were separated by SDS gel electrophoresis, transferred to nylon membranes and immunoblotted with antisera against Fhit [36], TK1 (Santa Cruz sc-134475), γH2AX (Millipore 05–636), and vinculin (Abcam ab18058).

### Immunofluorescence

Cells were grown on glass coverslips in 6 well plates (Fisher), fixed with 4% paraformaldehyde, permeabilized with 0.1% triton X, and blocked in 1% BSA. Slides were washed and coverslips mounted using Fluoro-Gel II with Dapi (Electron Microscope Sciences). Images were acquired at room temperature with an Olympus FV1000 spectral confocal microscope, a UPLFLN 100X objective lens, NA 1.30, and with Olympus FLOWVIEW acquisition software.

### Statistics

All p-values were determined from Mann Whitney U test.

## SUPPLEMENTARY FIGURE


