# Supplementary material for: FHIT loss-induced DNA damage creates optimal APOBEC substrates: Insights into APOBEC-mediated mutagenesis
Source: Oncotarget. 2014 Oct 31;6(5):3409–19. doi: 10.18632/oncotarget.2636 (PMC4413662; doi:10.18632/oncotarget.2636)
Supplement: Supplementary file 1 [file oncotarget-06-3409-s001.pdf]

SUPPLEMENTARY FIGURE

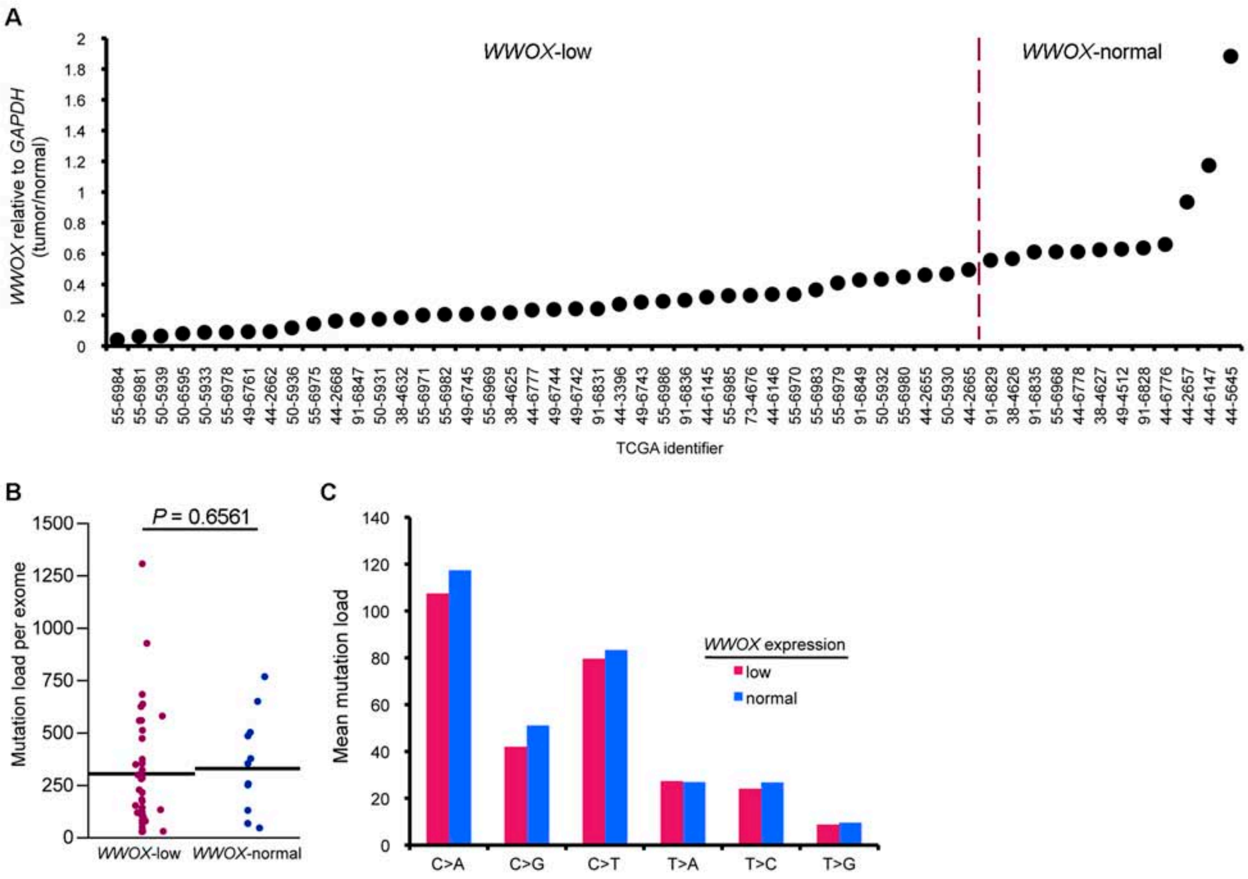

Supplementary Figure S1: Mutation analysis in *WWOX*-normal vs *WWOX*-low lung adenocarcinoma DNAs. (A) *WWOX* mRNA levels (tumor/normal) in the indicated TCGA lung adenocarcinoma samples. Tumor DNAs were grouped into *WWOX*-normal and *WWOX*-low lung tumor DNAs (>0.5 T/N-normal, <0.5 T/N-low). **(B)** Mutation load per exome in *WWOX*-normal and *WWOX*-low tumor DNAs. **(C)** Mean mutation load for specific point mutations in *WWOX*-normal and *WWOX*-low tumor DNAs. *P* value in (B) is from Mann-Whitney U test.
